# Orthorexia nervosa and dieting in a non-clinical sample: a prospective study

**DOI:** 10.1007/s40519-021-01353-8

**Published:** 2022-01-07

**Authors:** Caterina Novara, Susanna Pardini, Francesco Visioli, Nicola Meda

**Affiliations:** 1 Department of General Psychology, University of Padova, Via Venezia 8, 35131 Padova, Italy; 2 Department of Molecular Medicine, University of Padova, Viale G. Colombo 3, Padova, Italy; 3 IMDEA-Food, CEI UAM + CSIC, Carr. de Canto Blanco 8, E, Madrid, Spain; 4 Department of Medicine, University of Padova, Via Giustiniani 2, Padova, Italy

**Keywords:** Mental health, Young adults, Prospective study, Eating behaviour, Dietary pattern, Orthorexia nervosa

## Abstract

**Purpose:**

Orthorexia Nervosa (ON) is characterised by excessive attention to a dietary regimen perceived as healthy. A critical factor in the distinction between ON and other eating disorders (EDs) is the dichotomy of quality-versus-quantity of food intake. We investigated whether specific types of diet or dieting frequency are associated with orthorexic features, explored the overlap between ON and EDs symptoms, and examined which constructs are predictive of ON after 6 months.

**Methods:**

A total of 1075 students (75.1% female, mean age 20.9) completed a set of questionnaires assessing Orthorexia, Eating Disorders, Obsessions and Compulsions, Anxiety and Depression; 358 individuals (79.9 female, mean age 20.9) agreed to participate in the study and completed the same questionnaires after 6 months. Different regression models were defined to investigate our hypothesis.

**Results:**

Findings suggest that ON is associated with the number and type of diets followed over a lifetime. Moreover, participants with EDs, body dissatisfaction, or a dysfunctional idea of thinness are more likely to report a greater degree of ON features. After 6 months, the best predictors of ON characteristics are the same ON characteristics assessed at the first administration, with a significant role in the ideal of thinness.

**Conclusions:**

ON is more frequent in individuals with a previous diagnosis of EDs and in individuals who followed a restrictive diet or a vegan/vegetarian one; the number of lifetime diets, beliefs, and behaviors related to the ideals of thinness or body dissatisfaction is common features of ON. Moreover, considering that having ON features in the past is the best ON predictor in the present, we can presume that ON is a construct stable over time.

**Level of evidence:**

Level IV: Evidence obtained from multiple time series analysis such as case studies. (NB: Dramatic results in uncontrolled trials might also be regarded as this type of evidence).

**Supplementary Information:**

The online version contains supplementary material available at 10.1007/s40519-021-01353-8.

## Introduction

Orthorexia Nervosa (ON) is an unhealthy eating behavior characterised by an excessive concern for a healthy diet or food quality [1]. Although not officially considered a mental disorder, and its conceptualisation is controversial [2–5], some standard criteria for diagnosis of ON have been proposed and can be summarised as follows: (i) recurrent worry about healthy dieting or food quality; (ii) excessive focus on strict dietary rules and emotional consequences when transgressed [2]; and (iii) significant impairment of daily life activities and distress due to ON [5, 6]. ON prevalence rate varied widely in previous studies (from 1% to over 88%) due to the controversial definition or heterogenous assessment instruments [7, 8].

A central feature of the current debate on ON concerns the differentiation between a pathological condition and a healthy lifestyle that entails an interest in proper food choices and weight control through healthy behaviors. Excessive attention to a healthy lifestyle might have medical and psychological consequences [6], yet many such behaviors are widely promoted by mass media and often endorsed by Western societies [9, 10]. In addition, the overlap with some diagnoses recognised as Eating Disorders (EDs) (e.g., Anorexia Nervosa (AN) or Bulimia Nervosa (BN)) and Obsessive–Compulsive Disorder (OCD) [11] make ON a multi-faceted and evolving framework.

Much research has highlighted a significant intersection between ON and EDs [12]. Otherwise, the distinctions between EDs and ON imply the dichotomy of quantity-versus-quality: while individuals with EDs are more concerned about food intake, those with ON are worried about food quality [6]. Furthermore, weight reduction for health reasons has been underscored in the ON framework [13]. Some qualitative clinical studies on hospitalised individuals have highlighted how the ON is associated with a diet for weight loss or dictated by health problems [2, 14, 15]. In addition, a study on individuals who have AN and BN features showed a high prevalence of ON characteristics that tend to increase after treatment [16]. Regarding the non-clinical population, particular attention has been paid to groups considered at risk for ON (physicians, dieticians, medical/nutrition/dietetic’s students, and individuals who followed diets for moral/healthy reasons [13, 17–22]). It is also important to consider previous and current dietary habits, the type and reasons for dieting, and the overlap with past EDs to investigate similarities and differences between ON and EDs in the non-clinical population.

Most of the literature regarding ON administered the ORTO-15 questionnaire [23]. Even if a cutoff has been extrapolated, the questionnaire does not differentiate healthy from problematic eating patterns [24–26]. Because of this, reports on the relationships between age, gender, Body Mass Index (BMI), and ON characteristics have been inconsistent. Some studies have shown that ON is more common among young individuals [27–29], whereas others [23, 30] have reported that orthorexic characteristics are more likely to emerge along with age. Yet, other investigations did not highlight any significant relationship between age and ON [31–33]. Cross-national studies reported that ON is more common in women [28, 34–37], although the female gender is often predominant in cohorts [38]. Three studies have shown that ON is more frequent in men [23, 27, 39], while other authors reported no relationship between gender and ON [26, 33, 40–44]. Data on BMI are also inconsistent [38]: while some research highlighted no associations between BMI and ON [1, 23, 33, 35, 37, 39, 45–47], several authors reported that a high BMI is related to the risk of ON [1, 27, 31, 34, 36, 42, 48, 49], or that a low BMI is a predictor of ON features [47, 50, 51]. A recent review highlighted that the relationship between ON and BMI is not clear [38]. Thus, it might be essential to consider the role of BMI in ON with valid instruments and within more multifaceted framework related to diet, the presence of physical and psychological disorders, or constructs associated with EDs. The Eating Habits Questionnaire (EHQ) [52] has noteworthy psychometric properties. In particular, it is composed of three scales: Knowledge, Feelings, and Problems. Although it does not yet have a cutoff threshold, the Problems subscale seems to differentiate disordered eating from healthy eating, as it refers to the specific interferences and problems due to the adhesion to diet. In addition, the Problems subscale has shown a convergent validity with EDs symptoms, obsessive–compulsive symptoms, and depression [15, 24, 52].

Most of the literature published thus far has focused on samples of individuals that follow a specific diet and investigated the quality of the diets pursued and the motivations that led to this choice (moral in nature, health, or weight loss [22, 53]). These populations might have constantly been dieting or had undertaken numerous diets in their lives that could have exacerbated ON behavior, hence broadly explaining the incidence of ON.

This work aimed at assessing whether the frequency and type of diet are associated with orthorexic features, ascertain the ON and EDs overlap, and explore which of these constructs are predictive of ON characteristics after a time frame of 6 months. We addressed these issues from a perspective viewpoint to understand which aspects related to ON are pivotal in predicting the symptomatology of ON after some time. In line with Zikgraf et al., [24], we anticipated that the EDs-related symptomatology is more associated with functional impairments. We also expect the number and qualities of diets (i.e., vegetarians, vegans) to be associated with any ON construct [32, 54, 55].

## Methods

### Study cohort

All procedures were approved by the University of Padova Psychology Ethical Committee (Area 17) under the latest version of the Declaration of Helsinki, and participants provided written informed consent. Recruitment took place in Padova, a city in North-Eastern Italy, between October 2019 and June 2020. At the start of different teaching classes, we presented, either in person or via pre-recorded videos, the aims of the prospective study [56] to approximately 8,000 University of Padova’s students. The students were handed out a card containing an URL. By accessing the URL, students could provide their informed consent and participate in the study. Every 6 months, the participants were automatically asked by email to participate in another data collection. A total of 1,477 students agreed to participate in the study (18.4% response rate). Of this pool, 1,075 participants matched the target population characteristics (Italian native speaker students aged 18–30); participants were excluded if non-native speakers outside the previous age range or partially completed the questionnaires. No other inclusion or exclusion criteria were applied, and completed a demographic schedule and questionnaires based on the RedCap web application [57].

### Measures

Demographics schedule: the participants were asked to report their gender, age, BMI (calculated by self-reported height and weight), years of education, marital status, and occupation. Moreover, participants had to report if they had ever been diagnosed with a mental health condition or other medical disorder/illness/disease or if they had a family history of mental health issues. Participants were asked to report (a) how many diets they had followed in their lifetime; (b) prescribed diets, self-created, or inspired by a diet found on the internet or in books; (c) time since the last diet, and what type of diet (prescribed, self-created); (d) if they had any food intolerance and—if so—to what type of food; e) if they avoided a specific type of food and why; and d) if they had some physical illness (such as metabolic or gastroenterological issues).

The Beck Depression Inventory-II (BDI-II) is a 21-item self-report scale that assesses the severity of affective, cognitive, motivational, vegetative, and psychomotor features of depression; it has been thoroughly validated [58–61]. It is based on a four-point Likert scale, scored from 0 (absent) to 3 (severe), and total scores range from 0 to 63. The Italian version showed good internal consistency, considering a sample of university students, patients with depression, and a group of individuals extracted from the general population (0.80 < Cronbach's α < 0.87). The test–retest reliability across a period of a month was good (r = 0.76), as well as good convergent, divergent, and criterion validity [60]. Our sample maintained a high internal consistency (BDI-II α = 0.91).

The Beck Anxiety Inventory (BAI) [62–65] is a 21-item self-report that measures physiological and cognitive anxiety symptoms on a four-point Likert scale scored from 0 (not at all) to 3 (severely), and total scores range from 0 to 63. The Italian version showed good psychometric properties. In a sample of students, its internal consistency (Cronbach's α = 0.89) and the test–retest reliability were good as well as for construct validity [65]. In the present study, high internal consistency has been shown (α = 0.92).

The Obsessive–Compulsive Inventory-Revised (OCI-R) [66, 67] is an 18-item self-report questionnaire assessing the Obsessive–Compulsive Disorder (OCD) symptoms on a five-point Likert scale (Not at all, A little, Moderately, A lot and Extremely), and total scores range from 0 to 72. The questionnaire comprises six subscales (Washing, Ordering, Hoarding, Mental Neutralizing, Obsessing, and Checking) composing an additional final total score. The original version has good reliability and validity indices of the OCI-R, showing strong convergence with established measures of OCD, moderate to high internal consistency across the six subscales, and adequate to high test–retest stability. Regarding the Italian version [67], the confirmatory factor analysis showed the original six-factor structure. Moreover, a good internal consistency is confirmed (0.76 < Cronbach's α < 0.94). A 30-day test–retest reliability was good (0.76 < r < 0.99) and convergent, discriminant and criterion validity were acceptable [67]. In this study, the questionnaire’s acceptable internal consistency is highlighted both for the total (α = 0.88) and the subscales scores (Cronbach’s α ranging from 0.67—“Mental Neutralizing” and “Checking” subscales—to 0.86 “Obsessing”).

The Eating Disorder Inventory – 3 (EDI-3) [68–70] is a 91-item self-report questionnaire investigating symptoms of Eating Disorders and other related psychological features on a six-point Likert scale from 1 (Never) and 6 (Always). The EDI-3 is composed of twelve subscales. Some of them are related to EDs’ symptoms (i.e., Drive for Thinness, Bulimia, and Body Dissatisfaction) and are grouped in the “Eating Disorder Risk”. The other scales are about psychological features related to EDs (Low Self-Esteem, Personal Alienation, Interpersonal Insecurity, Interpersonal Alienation, Interoceptive Deficits, Emotional Dysregulation, Perfectionism, Asceticism, and Maturity Fears), and are grouped in the “General Maladjustment” scale. The present study categorised the participants in different manifestations of symptoms’ severity (as described in [68]) of eating disorders. For the General Maladjustment subscale, we considered that a score below 37 (25th percentile) means mild or absent symptomatology, whereas a score above 80 (66th percentile) marks the presence of severe symptoms. Between these two cutoffs, the score underlies moderate symptomatology. For the Eating Disorder Risk subscale, a score below 12 (25th percentile) is associated with mild or absent symptomatology, whereas a score above 36 (66th percentile) indicates the presence of severe symptoms. A score between 12 and 36 is associated with moderate symptomatology.

Good internal consistency was highlighted in the Italian version (0.72 < Cronbach’s α < 0.94) [69], as well as a good day test–retest reliability, cross-informant agreement, and a good discriminating validity [68, 69]. In the current study, Cronbach α was high for the total score and its subscales, i.e., total α = 0.96, General Maladjustment α = 0.95, and ED Risk α = 0.94).

The Eating Habits Questionnaire (EHQ) [52–71] is a 21-item self-report questionnaire aimed to assess ON characteristics on a four-point Likert scale from 1 to 4 (“false, not at all true,” “slightly true,” “mainly true,” and “very true”); total scores range from 0 to 84. It is characterised by three subscales (Knowledge, Feelings, and Problems), which refer to the negative impact that the participants’ diet style has had on the family, work, social and daily life (i.e., “I go out less since I began eating healthily”). The Italian validation, exploratory, and confirmatory factorial analysis evidence the same original EHQ structure [71]. Moreover, a good internal consistency and a 1-month test–retest reliability were highlighted (r ranging from 0.50 to 0.75; 0.001 < p < 0.01). The original and Italian versions highlighted adequate internal consistency indices and convergent and divergent validity [52, 71]. Based on the preliminary analysis conducted on Italian samples, including both groups of individuals with clinical problems and extracted from the general population, a score higher than 50 on the EHQ and corresponding to the 90th percentile could be considered helpful in discriminating individuals with high and low orthorexic features [15, 71]. In this study, the reliability was acceptable (for total score α = 0.87; Problems subscale α = 0.85; Knowledge subscale α = 0.76; Feelings subscale α = 0.64 after removing item 9—see also the Appendix).

## Statistical analysis

Anonymised data were downloaded from the REDCap [72] platform and curated using RStudio 3.5.3 [73–76]. Participants were divided into different severity classes according to their scores on the EDI-3 questionnaire [68, 69]; this is a practical approach for distinguishing the role of symptom severity on the studied outcome [68, 69].

In this work, we investigated the variables associated with characteristics of orthorexia with the EHQ. We analysed the factors associated with higher scores (i.e., more severe characteristics) as described below. To assess the association of the EHQ total and subscales scores with demographic factors and variables related to personal life, such as the history of mental disorders, eating behaviors, we defined different regression models (generalised linear mixed models, GLMMs). We used the Poisson family of distributions to model the data (as employed in [76–79] for count data with left-skewed distributions). Here we report only the models with the lowest Akaike Information Criterion (AIC—an index of model fitting: the lower the AIC, the lower the variance left unexplained by the model, the better the fit) identified with a stepwise selection approach. We report the β estimate for each variable of the model with the lowest AIC. The estimation of a variable represents the importance of that variable in changing the questionnaire scores. We ensured that scores were not better predictors of orthorexia symptomatology before categorising—symptom severity into classes. To assess what factors were associated with a relevant score (i.e., >  = 50) of the EHQ questionnaire, we defined a binomial regression model and applied the same principles of analysis described above for the other GLMMs.

Sample characteristics and regression models employed are reported in Tables 1 and 2 and in the Appendix, respectively.

**Table 1:** Sample characteristics

	Females w/out past ED	Females with past ED	Females (total)	Males	All participants
No. participants (%)	754 (93,3%)	54 (6,7%)	808 (75,1%)	267 (24,9%)	1075
Age (mean ± sd)	20.9 ± 2.03	21.1 ± 2.21	20.9 ± 2.04	21 ± 2.23	20.9 ± 2.09
Normal weight [No. (%)]	647 (85,9%)	39 (72,3%)	686 (85%)	214 (80,4%)	900 (83,8%)
Underweight [No. (%)]	55 (7,3%)	8 (14,8%)	63 (7,8%)	9 (3,3%)	72 (6,6%)
Overweight [No. (%)]	42 (5,5%)	6 (11,1%)	48 (5,9%)	40 (14,9%)	88 (8,2%)
Obese [No. (%)]	10 (1,3%)	1 (1,8%)	11 (1,3%)	4 (1,4%)	15 (1,4%)
EHQ Total (mean ± sd; median, [IQR])	40.8 ± 8.75; 40, [35-46]	50.8 ± 9.66; 51, [45-58.8]	41.5 ± 9.15; 40, [35-47]	39.5 ± 7.97; 38, [34-44]	41 ± 8.91; 40, [35-46]
EHQ knowledge	12.8 ± 3.08; 13, [11-15]	14.0 ± 3.07; 15, [13-16]	12.9 ± 3.09; 13, [11-15]	12.6 ± 2.94; 13, [10-15]	12.8 ± 3.06; 13, [11-15]
EHQ feelings	10.2 ± 2.59; 10, [9-12]	11.4 ± 2.07; 11, [10-12.8]	10.3 ± 2.57; 10, [9-12]	9.79 ± 2.32; 10, [8-11]	10.1 ± 2.52; 10, [9-12]
EHQ problems	17.8 ± 5.2; 16, [14-20]	25.4 ± 7.49; 25, [19-30.8]	18.3 ± 5.7; 17, [14-21]	17.2 ± 4.55; 16, [14-19]	18.0 ± 5.46; 16, [14-20]
Drive for thinness	8.45 ± 7.25; 6, [2-13]	15.4 ± 7.50; 16, [10-21]	8.91 ± 7.47; 7, [3-14]	4.36 ± 4.74; 3, [1-5]	7.78 ± 7.16; 5, [2-12]
Vegans [No. (%)]	7 (0,9%)	2 (3,7%)	9 (1,1%)	0	9 (0,8%)
Vegetarians [No. (%)]	39 (5,1%)	5 (9,2%)	44 (5,4%)	5 (1,8%)	49 (4,5%)
With anorexia nervosa [No. (%)]	//	//	39 (4,8%)	0	39 (3,6%)
With bulimia nervosa [No. (%)]	//	//	18 (2,2%)	0	18 (1,6%)
With a physical illness/disease/disorder [No. (%)]	121 (16%)	11 (20,3%)	132 (16,3%)	29 (10,8%)	161 (14,9%)
Followed at least one diet [No. (%)]	374 (49,6%)	51 (94,4%)	425 (52,5%)	96 (35,9%)	521 (48,4%)

Participants with a BMI lower than 18 have underweight. Participants with a BMI higher than 25.0 but lower than 30.0 are considered to have overweight. Participants with a BMI equal to or higher than 30 have at least Grade I Obesity

*ED* eating disorder(s), *EHQ* eating habits questionnaire, *IQR* interquartile range

**Table 2:** Characteristics, at t-zero, of the students that also participated in the 2nd administration

	Females w/out past ED	Females with past ED	Females (total)	Males	All participants
No. participants (%) to 2nd cross-sectional; characteristics at 1st cross-sectional	262 (91,6%)	24 (8,4 %)	286 (79,9%)	72 (20,1%)	358 (33,3% of 1^st^ cross-sectional participants)
Age (mean ± sd)	20.8 ± 2.09	21.2 ± 2.63	20.8 ± 2.14	21.1 ± 2.16	20.9 ± 2.14
Normal weight [No. (%)]	219 (83,8%)	17 (70,9%)	236 (82,6%)	63 (87,6%)	299 (83,7%)
Underweight [No. (%)]	24 (9,1%)	5 (20,8%)	29 (10,1%)	2 (2,7%)	31 (8,6%)
Overweight [No. (%)]	18 (6,8%)	2 (8,3%)	20 (7%)	6 (8,3%)	26 (7,2%)
Obese [No. (%)]	1 (0,3%)	0	1 (0,3%)	1 (1,4%)	2 (0,5%)
EHQ Total (mean ± sd; median, [IQR])	40.1 ± 8.59; 39, [34-45]	50.4 ± 9.24; 49, [43.8-58.2]	41 ± 9.09; 39, [34-46]	38.7 ± 7.4; 38, [33.8-43.2]	40.5 ± 8.82; 39, [34-46]
EHQ knowledge	12.7 ± 3.21; 13, [10-15]	14.1 ± 2.8; 14, [13-15.2]	12.8 ± 3.2; 13, [10.2-15]	12.5 ± 3.15; 13.5, [10-15]	12.7 ± 3.19; 13, [10-15]
EHQ feelings	10 ± 2.59; 10, [8-12]	11.4 ± 2.08; 11, [10-13]	10.1 ± 2.57; 10, [9-12]	9.78 ± 2.41; 10, [8-12]	10.1 ± 2.54; 10, [9-12]
EHQ problems	17.4 ± 5; 16, [14-19.8]	24.9 ± 7.18; 24.5, [18.8-31]	18.0 ± 5.6; 16, [14-21]	16.4 ± 3.69; 16, [14-18]	17.7 ± 5.31; 16, [14-20]
Drive for thinness	8.1 ± 7.09; 6, [2.25-12]	16 ± 7.84; 16.5, [10.8-23]	8.77 ± 7.47; 6, [3-13]	4 ± 3.94; 3, [1-5]	7.81 ± 7.17; 5, [2-12]
Vegans [No. (%)]	3 (1,1%)	2 (8,3%)	5 (1,7%)	0	5 (1,4%)
Vegetarians [No. (%)]	16 (6,1%)	3 (12,5%)	19 (6,6%)	1 (1,4%)	20 (5,5%)
With anorexia nervosa [No. (%)]	//	//	18 (6,3%)	0	18 (5%)
With bulimia nervosa [No. (%)]	//	//	7 (2,4%)	0	7 (1,9%)
With a physical illness/disease/disorder [No. (%)]	19 (7,2%)	5 (20,8%)	42 (14,6%)	6 (8,3%)	48 (13,4%)
Followed at least one diet [No. (%)]	124 (47,3%)	21 (87,5%)	145 (50,7%)	27 (37,5%)	172 (48,8%)

No male participants reported an eating disorder history. Participants with a BMI lower than 18 have underweight. Participants with a BMI higher than 25.0 but lower than 30.0 are considered to have overweight. Participants with a BMI equal to or higher than 30 have at least Grade I Obesity

*ED* eating disorder(s), *EHQ* eating habits questionnaire, *IQR* interquartile range

## Results

Of the 1,075 participants who completed the questionnaires at t-zero, 358 completed the questionnaires 6 months later. Students that participated in the study at t-zero were enrolled in Medicine and Surgery (37.8%), Science, Technology, Engineering, or Mathematics (21.9%), Biology, Pharmacy, or other Life Sciences (12.7%), Social and Political Sciences, or Law or Economics (10.7%), Psychology (9%), Arts and Humanities (7.9%). Students who were married or cohabiting were 2.7% (*n* = 29), while 97.3% were single or in a relationship. Only 3.2% of the participants worked (full-time or part-time or project work), whereas 96.8% were full-time students. As reported elsewhere [56], the lockdowns that followed the coronavirus pandemic did not significantly influence the EHQ scores in these participants.

The group of students who participated in the follow-up did not significantly differ in the prevalence of self-reported AN and BN from participants who did not participate in the follow-up. Moreover, we tested the prevalence of the disorders in the two groups at the first cross-sectional, knowing which participants would eventually participate or not in the second cross-sectional; AN: Pearson’s χ2 (1, *N* = 1075) = 3.009, *p* = 0.08; BN: Pearson’s χ2 (1, *N* = 1075) = 0.25, *p* = 0.61). However, the prevalence of history of major depressive disorder was higher in the group that took part in the second cross-sectional (Pearson’s χ2 (1, *N* = 1075) = 7.007, *p* = 0.008). Females percentage of participants was higher in the sample that completed the second wave than in the sample that participated only in the first wave (Pearson’s χ2 (1, *N* = 1075) = 6.42, *p* = 0.01). The comparisons for other relevant characteristics are not statistically significant and are reported in the Appendix.

### ON, diets and ED characteristics

For each subscale and the EHQ total score, we defined several regression models. We tested the role of each variable reported by the participants to determine what individual characteristics significantly altered the scores of the questionnaires, i.e., were associated with more severe characteristics.

**Fig. 1:**
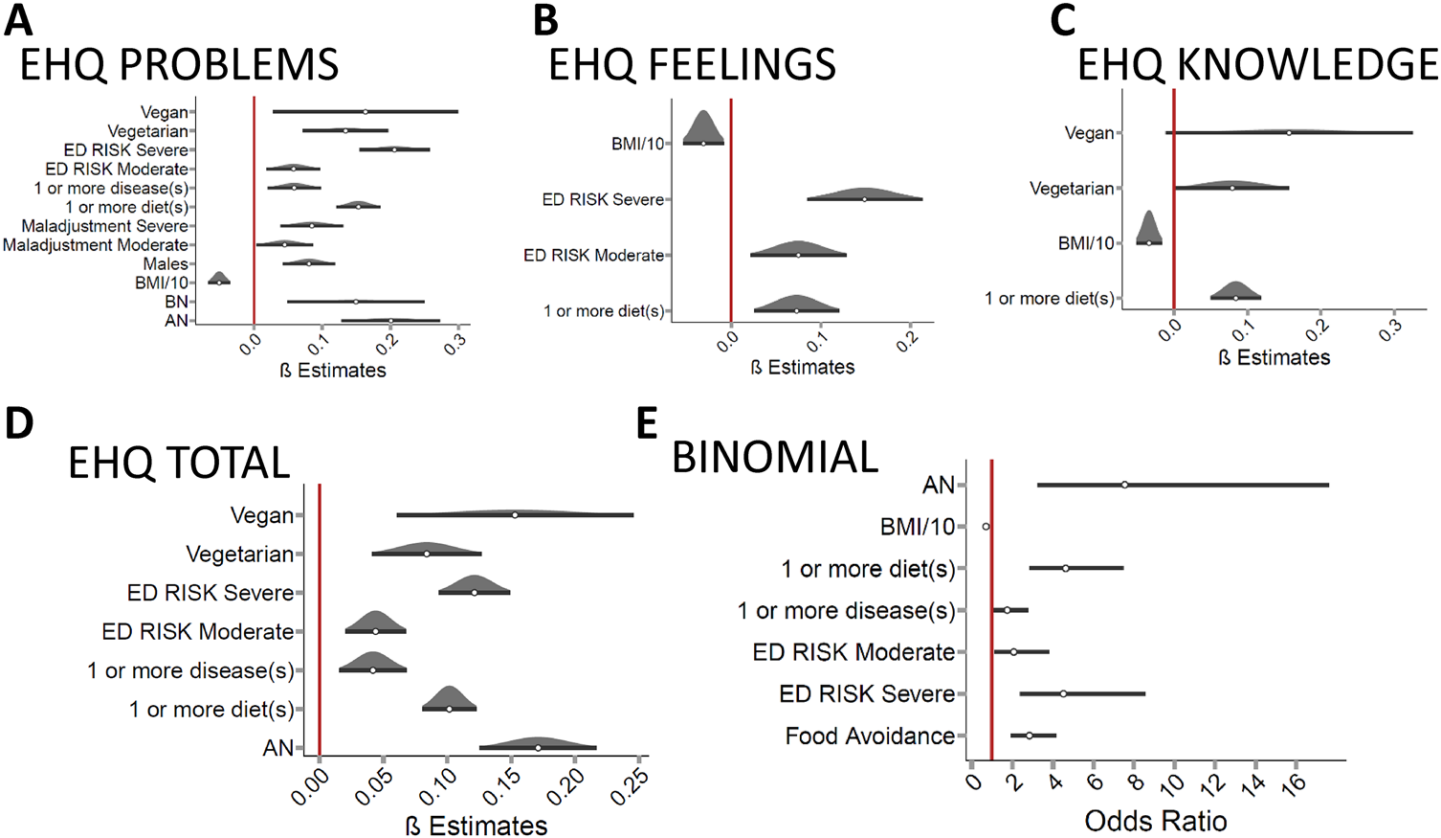
Factors associated with features of orthorexia nervosa. EHQ = eating habits questionnaire; ED RISK = classes of symptoms severity; Maladjustment = classes of symptoms severity BMI = body mass index; BN = bulimia nervosa; AN = anorexia nervosa. The red line represents the significance level. Factors at the left of the line are associated with a lower score, whereas factors at the right increase the score. Factors that were significantly associated with a higher score at the **A**EHQ Problems subscale. **B** EHQ Feelings subscale. **C**EHQ Knowledge subscale. **D** EHQtotal score. **E** Factors that increase the odds of having relevant features of orthorexia nervosa

Several factors were associated with higher scores at the Problems subscale (Fig. 1A and Appendix Table 1): commitment to a vegetarian/vegan diet was associated with more social, work or individual problems than being “omnivore” (vegetarian: β = 0.13, CI 95% (0.07–0.2), p < 0.001; vegan: β = 0.16, CI 95% (0.03–0.3), *p* < 0.05). Participants who had followed (or were following at the time of testing) at least one diet in their lifetime had more severe ON characteristics than those who had never followed any dietary regimen (β = 0.15, CI 95% (0.12–0.19), *p* < 0.001). Participants who self-reported a diagnosis of any physical illness, disease, or disorder were also more likely to have higher scores (β = 0.06, CI 95% (0.02–0.1), *p* < 0.01), as well as participants with a history of bulimia nervosa (β = 0.15, CI 95% (0.05–0.25), *p* < 0.001) or anorexia nervosa (β = 0.2, CI 95% (0.13–0.27), *p* < 0.001). We also evidenced a bias in the score against male participants, who suffered more severe ON characteristics than females (β = 0.08, CI 95% (0.04–0.12), *p* < 0.001), even though none of the male participants reported suffering or having suffered from an eating disorder. Among the other questionnaires, the EDI–III subscales (ED Risk and General Maladjustment) scores were significantly associated with higher scores at the EHQ Problems subscale (Maladjustment, moderate symptoms: β = 0.04, CI 95% (0.0–0.09), *p* < 0.05; severe symptoms: β = 0.08, CI 95% (0.04–0.13), *p* < 0.001. ED Risk, moderate symptoms: β = 0.06, CI 95% (0.02–0.1), *p* < 0.01; severe symptoms: β = 0.21, CI 95% (0.15–0.26), *p* < 0.001). The only factor associated with less severe symptomatology was BMI: for every 10 points increase of BMI, the score was reduced by β = − 0.17 (CI 95% − 0.23 to − 0.12, *p* < 0.001).

Feelings related to food and diet were more pronounced in participants who had more severe eating disorder symptoms, as measured by the scores on the ED Risk subscale (Fig. 1B and Appendix Table 2A. See Appendix Table 2B for model characteristics when also considering item 9 in the Feelings score. Moderate symptoms: β = 0.07, CI 95% (0.02–0.13), *p* < 0.01; severe symptoms: β = 0.15, CI 95% (0.08–0.21), *p* < 0.001), or in participants who reported having followed at least one diet in their lifetime (β = 0.07 CI 95% (0.03–0.12), *p* < 0.001). In addition, for this subscale, for every 10 points increase of BMI, the score was reduced by β = − 0.11 (CI 95% − 0.18 to − 0.03, *p* < 0.01).

Similar to the Problems subscale, also the Knowledge subscale scores were influenced by commitment to vegetarian diets (Fig. 1C and Appendix table 3. β = 0.08 CI 95% (0.0–0.16), p < 0.05) and non-significantly by vegan ones (β = 0.16 CI 95% (− 0.01–0.32), p = 0.067). Participants who followed at least one diet in their lifetime scored higher at this subscale (β = 0.08 CI 95% (0.05–0.12), p < 0.001). The negative impact of these factors was to some extent mitigated by the effect of BMI: for every ten units of BMI, the score was reduced by a factor of β = − 0.12, (CI 95% − 0.18 to − 0.06), *p* < 0.001.

Considering the EHQ Total score, the factors that influenced this score overlapped with those influencing the Problem subscale (Fig. 1D and Appendix Table 4). Commitment to a vegetarian (β = 0.08, CI 95% (0.04–0.13), *p* < 0.001) or vegan dietary regimen (β = 0.15, CI 95% (0.06–0.25), *p* < 0.01), or having followed at least 1 diet (β = 0.1, CI 95% (0.08–0.12), *p* < 0.001) were factors that increased the severity of manifestations of orthorexia. Moreover, a history of a physical illness, disease, or disorder (β = 0.04, CI 95% (0.02–0.07), *p* < 0.01) also increased the symptomatology, but not to the same extent as having suffered, or is suffering from AN (β = 0.17, CI 95% (0.13–0.22), *p* < 0.001). For the EHQ Total score, we report a significant association of the score with the severity of eating disorder symptoms as measured by the ED Risk subscale (moderate symptoms: β = 0.04, CI 95% (0.02–0.07), *p* < 0.001; severe symptoms: β = 0.12, CI 95% (0.09–0.15), *p* < 0.001)).

### ON, diets and ED characteristics in the high orthorexic sample

We defined different binomial regression models to evidence which factors increased the odds of experiencing clinically relevant manifestations of orthorexia (defined as an EHQ Total score ≥ 50; Fig. 1E, Appendix Table 5). As previously described for the EHQ Total score, the severity of eating disorder symptoms (measured with ED Risk subscale) was associated with a higher risk of experiencing relevant manifestations of ON (moderate symptoms: Odds Ratio = 2.07, CI 95% (1.13–3.8), *p* < 0.05; severe symptoms: OR = 4.52, CI 95% (2.39–8.54), *p* < 0.001)), a risk also conveyed by having followed at least one diet in the lifetime (OR = 4.64, CI 95% (2.87–7.47), *p* < 0.001), or reporting food avoidance, irrespective of the reason (OR = 2.84, CI 95% (1.93–4.18), *p* < 0.001). Have a physical illness, disease, or disorder was also associated with higher odds of experiencing relevant manifestations (OR = 1.75, CI 95% (1.09–2.81), *p* < 0.05), but a history of anorexia nervosa was the factor that increased the most the risk of experiencing relevant manifestations of orthorexia (OR = 7.55, CI 95% (3.24–17.62), *p* < 0.001). The only protective factor was BMI. For every ten units of BMI, OR = 0.3, CI 95% (0.14–0.64), *p* < 0.01).

### ON and ED characteristics in the sample with at least one diet

To further investigate the role of dieting, we analysed characterised different variables related to participants’ nutrition and dietetic choices. At t-zero, approximately half (48%, Table 1) of our sample with at least one diet in the lifetime.

**Fig. 2:**
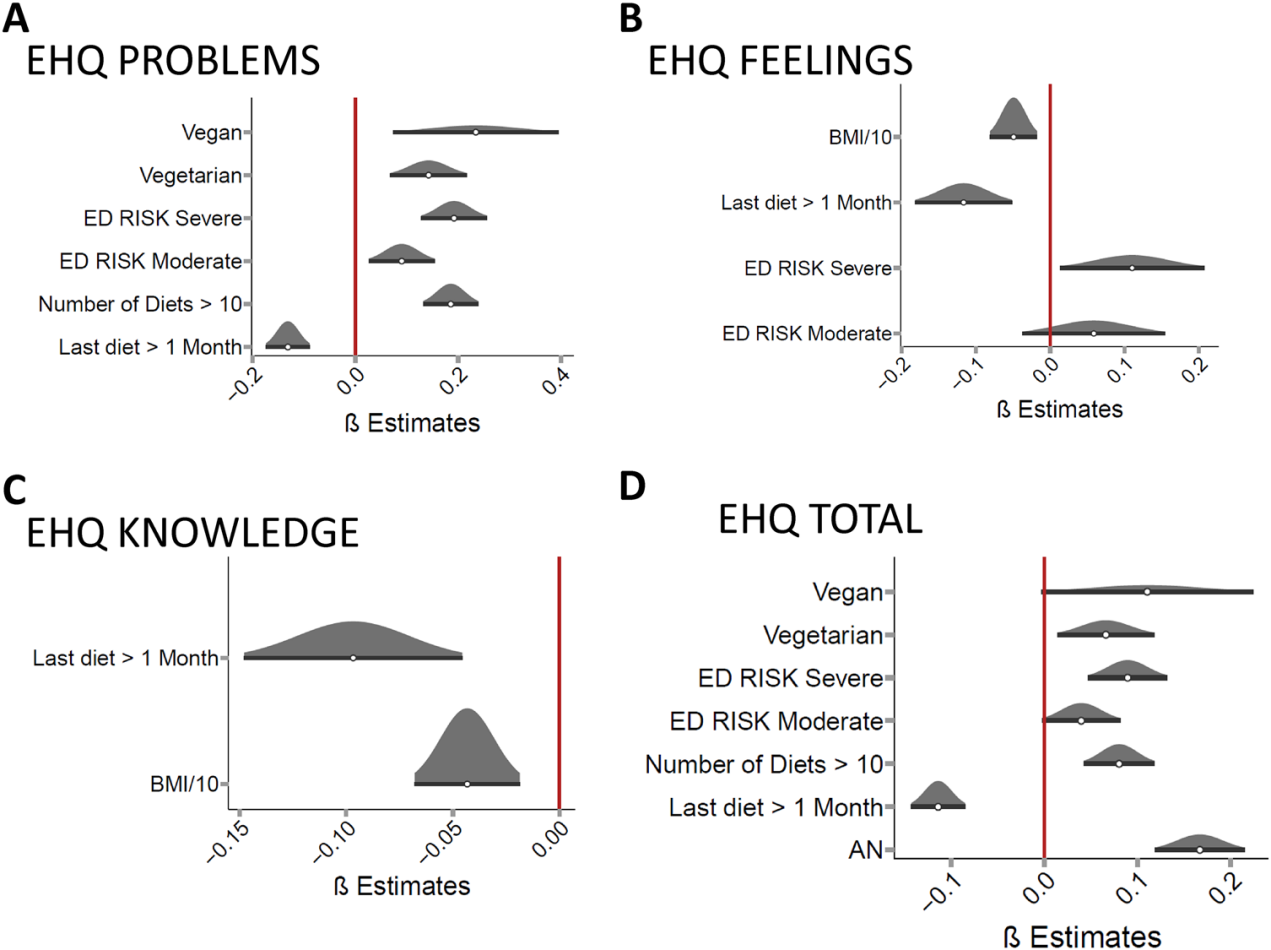
Orthorexia nervosa in participants with one diet in their lifetime. EHQ = eating habits questionnaire; ED RISK = classes of symptoms severity; BMI = body mass index; AN = anorexia nervosa. **A** Factors that were significantly associated with a higher score at the EHQ Problems subscale. **B** at the EHQ Feelings subscale. **C** at the EHQ Knowledge subscale. **D** at the EHQ total score

Participants who reported having followed more than ten diets had higher EHQ Problems scores (β = 0.19, CI 95% (0.13–0.24), p < 0.001) than participants who had followed less than ten diets (Fig. 2A, Appendix Table 6). Moreover, we found that participants who had followed a diet more than a month before taking the survey experienced fewer manifestations than those who followed a diet in the last month (β = − 0.13, CI 95% (− 0.17 – -0.09), *p* < 0.001). Commitment to a vegetarian or vegan diet and higher EDs symptom severity further increased the EHQ Problems score (Appendix Table 6).

Analogously, we found that having followed a diet more than a month before taking the survey resulted in a lower EHQ Feelings score than a more recent diet (β = − 0.12, CI 95% (− 0.18 to − 0.05), *p* < 0.001). Moreover, for every 10-point increase in the BMI, the score was reduced by a factor β = − 0.15 (CI 95% (− 0.24 to − 0.05)), *p* < 0.01), whereas the only other variable that significantly increased the manifestations measured by this subscale was the eating disorders symptoms severity (moderate: not significant, severe symptoms: β = 0.11, CI 95% (0.01–0.21), *p* < 0.05, Fig. 2B, Appendix Table 7A. See Appendix Table 7B for model characteristics when also considering item 9 in the Feelings score).

For the participants who reported having followed at least one diet, the characteristics measured by the EHQ Knowledge subscale could only be mitigated by two factors: BMI (for every ten units: β = − 0.13, CI 95% (− 0.2 to − 0.06), *p* < 0.001) and, as reported for the previous subscales, the time from the last diet (at least 1 month from the survey β = − 0.1, CI 95% (− 0.15 to − 0.05), *p* < 0.001, Fig. 2C, Appendix Table 8).

Regarding the characteristics of orthorexia measured by the EHQ Total score, participants who had followed more than ten diets in their lifetime reported higher scores than those who followed a smaller number of diets (β = 0.08, CI 95% (0.04–0.12), *p* < 0.001, Fig. 2D, Appendix Table 9). On the other hand, if the last followed diet dated to more than a month before the survey, the manifestations were somehow mitigated (β = − 0.11, CI 95% (− 0.14 to − 0.09), p < 0.001). Commitment to a vegetarian regimen further increased the EHQ Total score (β = 0.07, CI 95% (0.01–0.12), *p* < 0.001), whereas being vegan did not reach statistical significance. Participants who experienced severe symptoms of eating disorders (measured with ED Risk subscale) were also more likely to experience more manifestations of orthorexia (β = 0.09, CI 95% (0.05–0.13), *p* < 0.001, whereas moderate symptoms did not significantly influence the EHQ Total score). Participants who reported a history of AN also reported worse manifestations of orthorexia (β = 0.17, CI 95% (0.12–0.22), *p* < 0.001). Finally, we tested if this group of participants (who followed at least one diet in their lifetime) reported EHQ scores 6 months after the first survey completion that could be predicted with variables acquired at t-zero. However, the EHQ scores of this sub-sample could be best described by the same models reported for the overall sample (i.e., models considering the EHQ scores at t-zero).

### ON characteristics at 6 months

Six months after the first cross-sectional survey, participants were automatically re-contacted via email. A total of 358 participants (33,3% of the sample size of the first cross-sectional) agreed to participate in a second wave and completed all the questionnaires. We tested all the variables acquired at the first cross-sectional in predicting the EHQ scores 6 months later.

**Fig. 3:**
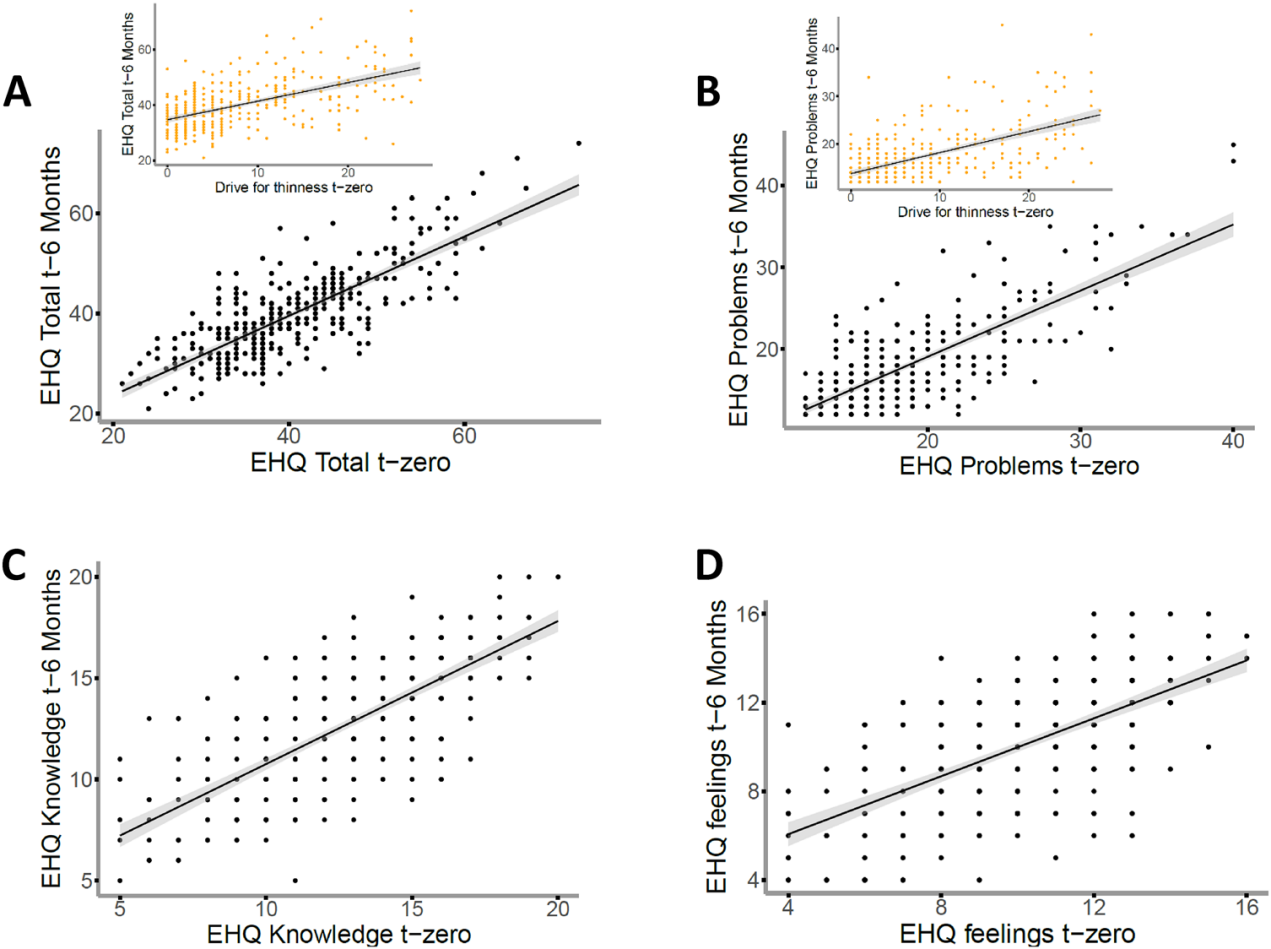
Predictors of Orthorexia Nervosa after 6 months. EHQ = Eating Habits Questionnaire; EHQ Total score (panel **A)**), EHQ Problems (panel **B)**), **C** Simple regression model of the EHQ Knowledge subscale **D** Simple regression model of the EHQ Feelings subscale

The best model to predict the EHQ Total score relies on the EHQ Total score itself and the drive for thinness at t-zero (Fig. 3A. For each point at the EHQ Total score at t-zero: β = 0.02, CI 95% (0.01–0.02), *p* < 0.001; for each point at the drive for thinness β = 0.003 CI 95% (0.00–0.01), *p* < 0.01; Marginal R^2^ of the model = 0.53).

Similarly, the EHQ Problems subscale score at 6 months is predicted by the EHQ Problems score and drive for thinness at t-zero (Fig. 3B. EHQ Problems score at t-zero: β = 0.04, CI 95% (0.03–0.04), *p* < 0.001; for each point at the drive for thinness β = 0.01 CI 95% (0.00–0.01), *p* < 0.01; Marginal R^2^ of the model = 0.45).

On the other hand, the EHQ Knowledge and EHQ Feelings scores can be best described by considering solely the Knowledge and Feelings scores at t-zero, respectively (Fig. 3C, D). EHQ Knowledge score at t-zero: β = 0.06, CI 95% (0.05–0.07), *p* < 0.001, Marginal R^2^ of the model = 0.30; EHQ Feelings score at t-zero: β = 0.07 CI 95% (0.05–0.08), *p* < 0.001; Marginal R^2^ of the model = 0.23).

## Discussion

It is of high clinical relevance to ascertain whether ON is a diagnostic category distinct from other eating disorders but characterized by specific dysfunctions in food-related behaviors. The current study aimed at extending our knowledge on ON by investigating its relationships with the amount and types of diets undertaken through a cross-sectional and longitudinal investigation. Dietary restrictions can be considered EDs’ risk factors [79] and are central elements in the ON hypothesis as a diagnostic category within eating disorders.

Our cross-sectional results have highlighted how the ON is dependent not only on the type (vegan and vegetarian) [13, 24] but also on the frequency of diet: having followed at least one diet in the lifetime is associated with more severe ON manifestations. Furthermore, as reported in other studies [34, 35, 80], our results confirm how the ON is related to the severity of EDs symptomatology linked to the dissatisfaction of one’s body and the ideal of thinness, and having had a diagnosis of anorexia nervosa.

Impairment of ON in daily life, based on the EHQ Problems score, was also associated with the type and number of diets, EDs, physical symptoms, and male gender. Even though none of the males reported a previous diagnosis of EDs, these results had been reported by other studies [81, 82]. The increasing focus of males on healthy lifestyles and diets could explain these data. Congruently, the knowledge about healthy diets was associated with dieting and diet type (i.e., vegan and vegetarian). The emotions about dieting were associated with dysfunctional beliefs on thinness and body dissatisfaction. Furthermore, our results show that higher BMI scores could be considered a protective factor from ON features: for every 10 points in the BMI, the EHQ score is 10 to 20% lower. Data could be interpreted as individuals with a lower BMI being more prone to dieting and weight control while also experiencing more significant concern and discomfort, impacting their global functioning. Overall, results confirm that ON is closely linked to AN’s presence in the past [16] and that pursuing diets and low BMI can be prodromes for eating problems.

The results obtained by considering the individuals with relevant orthorexic manifestations (EHQ > 90th percentile) further support our data: features related to EDs, diets, and diseases are significantly associated with relevant ON scores, whereas a higher BMI is a protective factor. While we expected a diagnosis of ED (including AN) to be associated with higher ON manifestations [36], having followed at least one diet during the lifetime (for any reason) and having EDs features (without an overt diagnosis) is equally associated with relevant ON characteristics. In this context, data could be due to participants having had EDs in the past or being at risk of developing EDs. Still, this piece of evidence might also point at the overlap of ON with body dissatisfaction, drive for thinness, and bulimic behaviors following several dietary restrictions.

Regarding participants who followed at least one diet in the past, our results showed that the number (> 10) and type of diet increase the ON manifestations and its consequences (Problems subscale). On the other hand, having stopped the diet for more than a month is considered a protective factor for ON; in this context, the presence of a higher BMI continues to be a protective factor. Participants following continuous dietary regimes may be at greater risk of developing ON and social and interpersonal problems resulting from dieting. Thus, it becomes pivotal to identify the number of diets conducted in the past and time elapsed from the last diet, considering the beneficial role of discontinuing diet on reducing ON.

Orthorexic features that emerged during the first wave were the best predictor of the ON characteristics after 6 months. Therefore, the orthorexic features do not change and are stable in time, supporting the hypothesis that ON can be considered a construct independent from EDs. The motivations of thinness represent the second-best predictor of ON: participants who endorse dysfunctional thinness ideals are more likely to experience psychological and interpersonal problems 6 months later.

No relationship was found with characteristics related to anxiety, depression, or obsessive and compulsive symptoms, confirming a part of the literature about the independence of the ON from these constructs [83–85].

## Conclusion

Our results confirm that ON is more represented in individuals with a past diagnosis of EDs [16], in individuals who pursue a restrictive diet or a vegan, vegetarian one [16, 22]. In addition, we have found that the number of diets, beliefs, and behaviors related to the ideals of thinness or body dissatisfaction are common features of ON. Moreover, considering that ON features assessed at t-zero are the best ON predictors after 6 months, we can presume that the ON construct is stable over time. All these aspects characterise the ON as a multi-faceted construct that could be considered a risk factor for the development or maintenance of EDs or could represent a problem per se, considering that ON is related to social or interpersonal impairments that remain constant time. Healthcare professionals should consider the dieting frequency during the lifetime as a possible link with the ON and with its problematic consequences. Moreover, it would be helpful to also consider orthorexic features in the assessment of the EDs.

### Strengths and limits

Regrettably, we have no information about the onset or duration of the physical or dietary problem, nor whether it has been resolved at the moment of questionnaire administration. Another limit concerns the second wave: about 1/3 of the individuals agreed to follow-up at 6 months. Even though there were no substantial differences in the demographic and psychological features between those who participated once or twice, the prospective evidence presented herein has to be taken with caution and confirmed with further studies. Moreover, the sample was composed of non-clinical Italian students and was mostly female: this poses an issue about the generalizability of our results. A wide array of validated questionnaires has been used to ascertain ON’s main contributors and benchmarks, but the assessment was based only on self-report questionnaires that ignore ego-syntonic characteristics. Furthermore, the EHQ does not assess negative emotions and concerns about eating unhealthy foods. Future studies should consider interviews or clinical judgments to overcome these problems.

### What is already known on the subject?

Orthorexia Nervosa (ON) is an unhealthy eating behavior characterised by an excessive concern for a healthy diet or food quality. Much research to date has highlighted a significant intersection between ON and ED. Still, among the distinctions, there is the dichotomy quantity-versus-quality of food intake that needs more investigation.

### What does this study add?

The number of diets, beliefs, and behaviors related to the ideals of thinness or body dissatisfaction are common features of ON. Moreover, considering that having ON features in the past is the best ON predictor in the present, we can presume that ON is a construct stable over time.

## Supplementary Information

Below is the link to the electronic supplementary material.

Supplementary file1 (DOCX 34 KB)

## Data Availability

The datasets used and analysed for the present study will be provided by the corresponding author upon reasonable request.
