# Supplementary material for: Orthorexia nervosa and dieting in a non-clinical sample: a prospective study
Source: Eat Weight Disord. 2022 Jan 7;27(6):2081–93. doi: 10.1007/s40519-021-01353-8 (PMC8739027; doi:10.1007/s40519-021-01353-8)
Supplement: Supplementary file 1 — Supplementary file1 (DOCX 34 KB) [file 40519_2021_1353_MOESM1_ESM.docx]

**Supplementary material**

**Orthorexia nervosa and dieting in a non-clinical sample: a prospective study**

**Novara,C., Pardini, S., Visioli, F., Meda N., (2021)**

**Contents**

Appendix Method………………………………………………………………………………………………2

Appendix Table 1..………………………………………………...……………………………………….…..4

Appendix Table 2A/2B..…………………………………………...……………………………………….…..5

Appendix Table 3..………………………………………………...……………………………………….…..6

Appendix Table 4..………………………………………………...……………………………………….…..7

Appendix Table 5..………………………………………………...……………………………………….…..8

Appendix Table 6..………………………………………………...……………………………………….…..9

Appendix Table 7A/B……………………………………………...……………………………………….…..10

Appendix Table 8..………………………………………………...……………………………………….…..11

Appendix Table 9..………………………………………………...……………………………………….…..12

**Appendix Method**

We defined different regression models to assess the association between the EHQ Total and EHQ subscales score with demographic variables and other questionnaires scores (generalised linear mixed models – GLMMs, below). We undertook a stepwise selection approach for the modelling: we evaluated the contribution of each relevant independent variable to the model and kept the variables that significantly improved the model. Since psychometric test scores are counting variables, we used the Poisson family of distributions.

(GLMM1) $\begin{aligned} \\ \begin{aligned} \begin{aligned} \mu\\ log\left( ij \right)=\beta_{0}+u_{ij} \\ \end{aligned} \end{aligned} \end{aligned}$

(GLMM2) $\begin{aligned} \\ \begin{aligned} \begin{aligned} \mu\\ log\left( ij \right)=\beta_{0}+\beta_{1}D_{1ij}+\ldots+\beta_{n}D_{nij}+u_{ij} \\ \end{aligned} \end{aligned} \end{aligned}$

Where:

$\mu_{ij}$ = incidence rate (i.e., the EHQ total (or subscales) score)

${D_{1ij}}/{D_{nij}}$ = 1^st^/nth categorical predictors

$u_{ij}$ = random effects refer to the variation of the intercepts among participants’ age (for first cross-sectional analyses) or for each participant (controlling for correlation of paired data when testing for longitudinal changes in the scores).

The models (e.g., GLMM1 and GLMM2) were compared using the *anova* function, and the model with the lowest Akaike Information Criterion (AIC) was selected.

To assess which factors were significantly associated with clinically relevant symptomatology (i.e., a score >= 50 in the EHQ questionnaire), we used a binomial (logistic) regression, structure analogously to the GLMM described above, in which the dependent variable ($\mu_{ij}$) was either 0 (no clinical relevance) or 1 (clinical relevance of the score).

We assessed if any demographic characteristics (e.g., prevalence of a particular disorder) or questionnaires scores were different between the group of participants who take part in the second cross-sectional and the group who did not. Score differences are tested with unpaired, two-samples Wilcoxon Test (normality assumption rejected after Shapiro-Wilk test); prevalence differences are tested with Pearson’s Chi square test.

|  | **Pearson’s Chi square test**  **(degrees of freedom = 1)** |  |
| --- | --- | --- |
| **Prevalence of any physical illness/disorder/disease** | χ^2^ = 1.037, p = 0.30 |  |
| **Prevalence of people who followed at least 1 diet** | χ^2^ = 0.037, p = 0.84 |  |
|  | **Shapiro-Wilk Normality test, group who did not take part in the 2^nd^ wave** | **Wilcoxon Test** |
| **Age** | W = 0.90, p < 0.0001 | W = 133265, p = 0.29 |
| **BMI** | W = 0.86, p < 0.0001 | W = 137242, p = 0.063 |
| **EHQ Total Score** | W = 0.96, p < 0.0001 | W = 133521, p = 0.28 |
| **EHQ Problems Score** | W = 0.85, p < 0.0001 | W = 135615, p = 0.12 |
| **EHQ Feelings Score** | W = 0.97, p < 0.0001 | W = 130303, p = 0.68 |
| **EHQ Knowledge Score** | W = 0.98, p < 0.0001 | W = 129457, p = 0.81 |
| **Drive for Thinness Score** | W = 0.88, p < 0.0001 | W = 126567, p = 0.71 |

The Body Mass Index (BMI) of participants who participate in the second wave tends to be lower (mean = 21.1, standard deviation = 2.5, median = 20.83, interquartile range = 3.1) than the BMI of the students who did not complete the second cross-sectional (mean = 21.5, standard deviation = 3.1, median = 21.05, interquartile range = 3.5).

|  | **EHQ Problems** |  |  | **EHQ Problems** |
| --- | --- | --- | --- | --- |
| *Predictors* | β Estimate (CI 95%) |  | *Predictors* | β Estimate (CI 95%) |
| (Intercept) | 3.00 ^***^ (2.88 – 3.11) |  | 1 or more diet(s) [Ref: None] | 0.15 ^***^ (0.12 – 0.19) |
| Anorexia Nervosa: Yes [Ref: No] | 0.20 ^***^ (0.13 – 0.27) |  | ED Risk Severity: Moderate [Ref: Mild/No] | 0.06 ^**^ (0.02 – 0.10) |
| Bulimia Nervosa: Yes [Ref: No] | 0.15 ^**^ (0.05 – 0.25) |  | ED Risk Severity: Severe [Ref: Mild/No] | 0.21 ^***^ (0.15 – 0.26) |
| BMI/10 | -0.17 ^***^ (-0.23 – -0.12) |  | Vegetarian [Ref: Omnivore] | 0.13 ^***^ (0.07 – 0.20) |
| Sex: Male [Ref: Female] | 0.08 ^***^ (0.04 – 0.12) |  | Vegan [Ref: Omnivore] | 0.16 ^*^ (0.03 – 0.30) |
| Maladjustment Severity: Moderate [Ref: Mild/No] | 0.04 ^*^ (0.00 – 0.09) |  | 1 or more disease(s) [Ref: None] | 0.06 ^**^ (0.02 – 0.10) |
| Maladjustment Severity: Severe [Ref: Mild/No] | 0.08 ^***^ (0.04 – 0.13) |  |  |  |
| Observations | 1075 |  |  |  |
| R^2^ | 0.323 |  |  |  |
| ** p<0.05   ** p<0.01   *** p<0.001* | | | | |

**Appendix Table 1.** **Variables of the Poisson regression model that describes the EHQ Problems score**. Ref = reference value from which the β Estimate for all other values of the same variable are computed (e.g., β Estimate of “Vegetarian” and “Vegan” is computed relative to “Omnivore”).; ED = Eating disorder; In the case of a continuous predictor (as the BMI/10), the estimate shall be multiplied with the value of the predictor. A positive estimate signifies a higher EHQ Problems score.

|  | | **EHQ Feelings** |  | **EHQ Feelings** |  |
| --- | --- | --- | --- | --- | --- |
| *Predictors* | | β Estimate (CI 95%) |  | β Estimate (CI 95%) |  |
| (Intercept) | | 2.18 ^***^ (2.02 – 2.34) | ED Risk Severity: Severe [Ref: Mild/No] | 0.15 ^***^ (0.08 – 0.21) |  |
| 1 or more diet(s) [Ref: None] | | 0.07 ^**^ (0.03 – 0.12) | BMI/10 | -0.11 ^**^ (-0.18 – -0.03) |  |
| ED Risk Severity: Moderate [Ref: Mild/No] | | 0.07 ^**^ (0.02 – 0.13) |  |  |  |
| Observations | | 1075 |  |  |  |
| R^2^ | | 0.045 |  |  |  |
|  | ** p<0.05   ** p<0.01   *** p<0.001* | | | |  |

**Appendix Table 2A.** **Variables of the Poisson regression model that describes the EHQ Feelings score when excluding item 9 (thus Cronbach’s α of the subscale = 0.64).** Ref = reference value from which the β Estimate for all other values of the same variable is computed (e.g., β Estimate of ED RISK severity “Moderate” and “Severe” is computed relative to “Mild/No”).

|  | **EHQ Feelings** |  |  | **EHQ Feelings** |  |
| --- | --- | --- | --- | --- | --- |
| *Predictors* | β Estimate (CI 95%) |  | *Predictors* | β Estimate (CI 95%) |  |
| (Intercept) | 2.18 ^***^ (2.14 – 2.22) |  | ED Risk Severity: Moderate [Ref: Mild/No] | 0.10 ^***^ (0.05 – 0.15) |  |
| 1 or more diet(s) [Ref: None] | 0.09 ^***^ (0.05 – 0.13) |  | ED Risk Severity: Severe [Ref: Mild/No] | 0.17 ^***^ (0.12 – 0.23) |  |
| Observations | 1075 |  |  | |  |
| R^2^ | 0.086 |  |  | |  |
| ** p<0.05   ** p<0.01   *** p<0.001* | | | | |  |

**Appendix Table 2B.** **Variables of the Poisson regression model that describes the EHQ Feelings score when including item 9 (thus Cronbach’s α of the subscale = 0.58).** Ref = reference value from which the β Estimate for all other values of the same variable is computed (e.g., β Estimate of ED RISK severity “Moderate” and “Severe” is computed relative to “Mild/No”).

|  | **EHQ Knowledge** |  | **EHQ Knowledge** |  |
| --- | --- | --- | --- | --- |
| *Predictors* | β Estimate (CI 95%) | *Predictors* | β Estimate (CI 95%) |  |
| (Intercept) | 2.75 ^***^ (2.62 – 2.88) | 1 or more diet(s) [Ref: None] | 0.08 ^***^ (0.05 – 0.12) |  |
| Vegetarian [Ref: Omnivore] | 0.08 ^*^ (0.00 – 0.16) | BMI/10 | -0.12 ^***^ (-0.18 – -0.06) |  |
| Vegan [Ref: Omnivore] | 0.16  (-0.01 – 0.32) |  |  |  |
| Observations |  | 1075 | |  |
| R^2^ |  | 0.038 | |  |
| ** p<0.05   ** p<0.01   *** p<0.001* | | | |  |

**Appendix Table 3.** **Variables of the Poisson regression model that describes the EHQ Knowledge score**

|  | **EHQ Total** |  | **EHQ Total** |  |
| --- | --- | --- | --- | --- |
| *Predictors* | β Estimate (CI 95%) | *Predictors* | β Estimate (CI 95%) |  |
| (Intercept) | 3.59 ^***^ (3.57 – 3.61) | ED Risk Severity: Moderate [Ref: Mild/No] | 0.04 ^***^ (0.02 – 0.07) |  |
| Anorexia Nervosa: Yes [Ref: No] | 0.17 ^***^ (0.13 – 0.22) | ED Risk Severity: Severe [Ref: Mild/No] | 0.12 ^***^ (0.09 – 0.15) |  |
| 1 or more diet(s) [Ref: None] | 0.10 ^***^ (0.08 – 0.12) | Vegetarian [Ref: Omnivore] | 0.08 ^***^ (0.04 – 0.13) |  |
| 1 or more disease(s) [Ref: None] | 0.04 ^**^ (0.02 – 0.07) | Vegan [Ref: Omnivore] | 0.15 ^**^ (0.06 – 0.25) |  |
| Observations | 1075 |  |  |  |
| R^2^ | 0.296 |  |  |  |
|  | ** p<0.05   ** p<0.01   *** p<0.001* | | |  |

**Appendix Table 4.** **Variables of the Poisson regression model that describes the EHQ Total score**.

|  | **Clinical Relevance** |  | **Clinical Relevance** |  |
| --- | --- | --- | --- | --- |
| *Predictors* | Odds Ratios (CI 95%) | *Predictors* | Odds Ratios (CI 95%) |  |
| (Intercept) | 0.18 ^*^ (0.03 – 0.92) | ED Risk Severity: Moderate [Ref: Mild/No] | 2.07 ^*^ (1.13 – 3.80) |  |
| 1 or more diet(s) [Ref: None] | 4.64 ^***^ (2.87 – 7.47) | ED Risk Severity: Severe [Ref: Mild/No] | 4.52 ^***^ (2.39 – 8.54) |  |
| 1 or more disease(s) [Ref: None] | 1.75 ^*^ (1.09 – 2.81) | BMI/10 | 0.30 ^**^ (0.14 – 0.64) |  |
| Anorexia Nervosa: Yes [Ref: No] | 7.55 ^***^ (3.24 – 17.62) | Food Avoidance | 2.84 ^***^ (1.93 – 4.18) |  |
| Observations | 1075 |  |  |  |
| Marginal R^2^ | 0.382 |  |  |  |
|  | ** p<0.05   ** p<0.01   *** p<0.001* | | |  |

**Appendix Table 5.** **Variables of the binomial regression model that describe the presence or absence of clinical relevance of the EHQ Total Score**. Ref = reference value from which the Odds Ratio for all other values of the same variable is computed (e.g., Odds Ratio of ED RISK severity “Moderate” and “Severe” is computed relative to “Mild/No”). A clinically relevant score of the EHQ Total scale is defined as equal to or above 50. Each variable value (e.g., Anorexia Nervosa: Yes) odds ratio describes the risk of having a clinically relevant score relative to the reference condition (e.g., Anorexia Nervosa: No).

|  | **EHQ Problems** |  | **EHQ Problems**  β Estimate (CI 95%) | | |
| --- | --- | --- | --- | --- | --- |
| *Predictors* | β Estimate (CI 95%) | *Predictors* |  |  |  |
| (Intercept) | 2.93 ^***^ (2.86 – 3.00) | Number of diets followed: >= 10 [Ref: < 10] | 0.19 ^***^ (0.13 – 0.24) | | |
| ED Risk Severity: Moderate [Ref: Mild/No] | 0.09 ^**^ (0.03 – 0.15) | Vegetarian [Ref: Omnivore] | 0.14 ^***^ (0.07 – 0.22) | | |
| ED Risk Severity: Severe [Ref: Mild/No] | 0.19 ^***^ (0.13 – 0.26) | Vegan [Ref: Omnivore] | 0.23 ^**^ (0.07 – 0.40) | | |
| Time from last diet > 1 Month [Ref: <= 1 Month] | -0.13 ^***^ (-0.17 – -0.09) |  |  | | |
| Observations | 521 |  |  |  | |
| R^2^ | 0.275 |  |  |  | |
| ** p<0.05   ** p<0.01   *** p<0.001* | | | | |  |

**Appendix Table 6.** **Variables of the Poisson regression model that describe the EHQ Problems score in the sample that reported having followed at least one diet in their lifetime**.

|  | **EHQ Feelings** |  | **EHQ Feelings** |
| --- | --- | --- | --- |
| *Predictors* | β Estimate (CI 95%) | *Predictors* | β Estimate (CI 95%) |
| (Intercept) | 2.45 ^***^ (2.23 – 2.68) | Time from last Diet > 1 Month [Ref: <= 1 Month] | -0.12 ^***^ (-0.18 – -0.05) |
| ED Risk Severity: Moderate [Ref: Mild/No] | 0.06  (-0.04 – 0.15) | BMI/10 | -0.15 ^**^ (-0.24 – -0.05) |
| ED Risk Severity: Severe [Ref: Mild/No] | 0.11 ^*^ (0.01 – 0.21) |  |  |
| Observations | 521 |  |  |
| R^2^ | 0.054 |  |  |
| ** p<0.05   ** p<0.01   *** p<0.001* | | | |

**Appendix Table 7A.** **Variables of the Poisson regression model that describe the EHQ Feelings score in the sample that reported having followed at least one diet in their lifetime**. **(excluding item 9, thus Cronbach’s α of the subscale = 0.64).**

|  | **EHQ Feelings** |  | | **EHQ Feelings** |
| --- | --- | --- | --- | --- |
| *Predictors* | β Estimate (CI 95%) | *Predictors* | β Estimate (CI 95%) | |
| (Intercept) | 2.38 ^***^ (2.29 – 2.47) | ED Risk Severity: Moderate [Ref: Mild/No] | | 0.07  (-0.01 – 0.16) |
| Time from last Diet > 1 Month [Ref: <= 1 Month] | -0.11 ^***^ (-0.17 – -0.05) | ED Risk Severity: Severe [Ref: Mild/No] | | 0.12 ^**^ (0.04 – 0.21) |
| Observations | 521 |  | |  |
| R^2^ | 0.051 |  | |  |
| ** p<0.05   ** p<0.01   *** p<0.001* | | | | |

**Appendix Table 7B.** **Variables of the Poisson regression model that describe the EHQ Feelings score in the sample that reported having followed at least one diet in their lifetime**. **(including item 9, thus Cronbach’s α of the subscale = 0.58).**

|  | **EHQ Knowledge** |
| --- | --- |
| *Predictors* | β Estimate (CI 95%) |
| (Intercept) | 2.94 ^***^ (2.77 – 3.11) |
| BMI/10 | -0.13 ^***^ (-0.20 – -0.06) |
| Time from last Diet > 1 Month [Ref: <= 1 Month] | -0.10 ^***^ (-0.15 – -0.05) |
| Observations | 521 |
| R^2^ | 0.048 |
| ** p<0.05   ** p<0.01   *** p<0.001* | |

**Appendix Table 8.** **Variables of the Poisson regression model that describe the EHQ Knowledge score in the sample that reported having followed at least one diet in their lifetime**.

|  | **EHQ Total** |  | **EHQ Total** |
| --- | --- | --- | --- |
| *Predictors* | β Estimate (CI 95%) | *Predictors* | β Estimate (CI 95%) |
| (Intercept) | 3.78 ^***^ (3.74 – 3.83) | Time from last Diet > 1 Month [Ref: <= 1 Month] | -0.11 ^***^ (-0.14 – -0.09) |
| Anorexia Nervosa: Yes [Ref: No] | 0.17 ^***^ (0.12 – 0.22) | Number of diets followed: >= 10 [Ref: < 10] | 0.08 ^***^ (0.04 – 0.12) |
| ED Risk Severity: Moderate [Ref: Mild/No] | 0.04  (-0.00 – 0.08) | Vegetarian [Ref: Omnivore] | 0.07 ^*^ (0.01 – 0.12) |
| ED Risk Severity: Severe [Ref: Mild/No] | 0.09 ^***^ (0.05 – 0.13) | Vegan [Ref: Omnivore] | 0.11  (-0.00 – 0.22) |
| Observations | 521 |  |  |
| R^2^ | 0.287 |  |  |
| ** p<0.05   ** p<0.01   *** p<0.001* | | | |

**Appendix Table 9.** **Variables of the Poisson regression model that describe the EHQ Knowledge score in the sample that reported having followed at least one diet in their lifetime**.
